# Entangled fingerprints for quantum-encoded chemoinformatics: quantum circuits for molecular similarity in the noisy era

**DOI:** 10.3389/fchem.2025.1707409

**Published:** 2026-01-06

**Authors:** Sergey Shityakov, Thomas Dandekar

**Affiliations:** 1 Department of Bioinformatics, Biocenter, University of Würzburg, Würzburg, Germany; 2 Laboratory of Chemoinformatics, Infochemistry Scientific Center, ITMO University, Saint Petersburg, Russia

**Keywords:** extended-connectivity fingerprints, near-term quantum computing, noise mitigation, quantum-encoded chemoinformatics, Tanimoto similarity

## Abstract

Quantum computing holds promise for molecular similarity analysis in chemoinformatics and drug discovery. We propose a quantum circuit to encode the classically pre-computed Tanimoto similarity (T), obtained from extended-connectivity fingerprints (ECFPs) with RDKit, into a compact three-qubit entangled state using Qiskit. A 3-qubit circuit with RY rotations encodes T coefficients, while CNOT gates create an entangled three-qubit state that serves as a sensitive probe for quantum noise and error-mitigation performance. Simulations under noise demonstrate that exponential mitigation reduces errors by 75.0% for similar pairs (e.g., aspirin–aspirin) and 87.5% for dissimilar pairs (e.g., aspirin–butane) at a 1% error rate, maintaining fidelity within ±0.001 deviation. At 10% depolarization noise, error reduction drops to 25.0% and 17.4% for these pairs, respectively. The overall results show that the mitigation is proportionally more effective for low-similarity pairs. Experiments on IBM Quantum hardware confirm Z-basis reliability but reveal challenges with X-basis noise. Our work demonstrates quantum-encoded T representation and recovery on NISQ devices as a proof-of-concept, highlighting the critical role of error mitigation in hybrid quantum-classical workflows.

## Graphical Abstract

**Figure:**
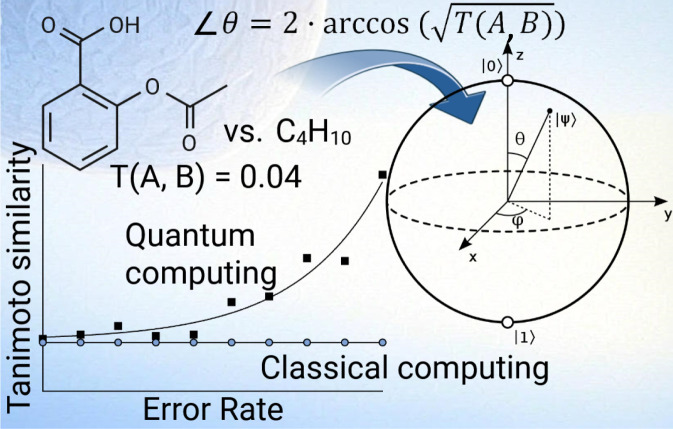

## 1 Introduction

Molecular similarity is a cornerstone of chemoinformatics and plays a vital role in virtual screening, compound clustering, and drug discovery (Isakova et al., 2024; López-Pérez et al., 2024; Shityakov and Dandekar, 2010). Quantifying the likeness between chemical compounds is crucial for predicting their biological activity and identifying potential drug candidates. Among the various metrics available, the Tanimoto similarity (*T*) coefficient, also known as the Jaccard index, stands out as a widely adopted and effective measure for comparing compounds on the basis of their binary fingerprint representations (Safizadeh et al., 2021).

Fingerprints, such as extended-connectivity fingerprints (ECFPs) or Morgan fingerprints, encode structural information into bit strings, allowing for rapid *T* calculations. These classical methods have been extensively used in computer-aided drug design, as described by Zhou and Skolnick (2024), and Shityakov et al. (2021) and provide a benchmark for novel computational approaches.

On the other hand, quantum computing presents an intriguing alternative for tackling complex problems in biochemistry and rational drug design. It offers the potential to leverage quantum parallelism to process probabilistic states more efficiently than classical counterparts in some domains.

Various studies have explored the application of quantum algorithms in computational chemistry, aiming to leverage quantum phenomena such as superposition and entanglement to improve performance (Biamonte et al., 2017; Mazzola, 2024; Quinton et al., 2025). For example, researchers have investigated quantum machine learning models for predicting molecular properties and activities, with some studies showing promising results in accuracy and efficiency compared with classical methods (Akrom et al., 2024). This study builds upon the integration of quantum chemical property features to reduce the computational burden while maintaining prediction accuracy for the corrosion inhibition of quinoxaline compounds (Akrom et al., 2024). Some *T* functions have also been implemented in sophisticated computational techniques to assess the degree of similarity between different images, optimizing the performance of quantum retrieval systems (Yang et al., 2025).

On the other hand, the application of quantum circuits to encode Tanimoto similarity via molecular similarity-based descriptors remains unexplored, particularly concerning how quantum noise affects the accuracy of *T* estimation. Given that quantum computers are still in their early stages of development and are susceptible to noise, understanding and mitigating the effects of noise is critical for realizing the full potential of quantum algorithms in chemistry (Prasad et al., 2024; Ziems et al., 2025). Therefore, this work contributes to quantum-encoded chemoinformatics by exploring the feasibility and limitations of quantum circuits for molecular similarity analysis. We investigated noise mitigation techniques to improve practical applicability, supporting hybrid quantum-classical workflows.

To our knowledge, this is the first demonstration of a compact 3-qubit quantum circuit that encodes a pre-calculated classical Tanimoto coefficient (derived from extended-connectivity fingerprints) into an entangled GHZ-like state. Although Tanimoto similarity itself is computed classically with maximal efficiency, this encoding provides a chemically motivated, minimal benchmark for rigorously evaluating noise resilience and error-mitigation strategies on current NISQ hardware.

## 2 Computational methods

We implemented RDKit v.2025.03.2 to compute ECFPs, specifically Morgan fingerprints) for input SMILES strings (Bento et al., 2020; Rogers and Hahn, 2010). The canonical SMILES for aspirin (PubChem CID: 2244) and butane (PubChem CID: 7843) was retrieved from the PubChem database (Cincilla et al., 2010). Equations 1–5 define the Tanimoto similarity and its quantum encoding. Equations 6–13 describe noise modeling, mitigation, and error metrics. The Tanimoto similarity between two fingerprint vectors was calculated as the ratio of the intersection over the union of their bits, defined as

$$T\left(A,B\right)=\frac{\left|A\cap B\right|}{\left|A\cup B\right|}$$ (1)

where A and B are the binary fingerprint vectors for two molecules, $\left|A\cap B\right|$ is the number of common bits set to 1, and $\left|A\cup B\right|$ is the total number of bits set to 1 in either vector.

The drug-likeness scores were calculated using the MolSoft drug-likeness model leverages a machine learning approach based on Naive Bayes (NB) classifier (MolSoft ICM-Pro v.3.9-2b, MolSoft LLC, academic license) according to the standard protocol (Poola et al., 2023).

The model incorporates a set of 64 physicochemical and topological descriptors, including molecular weight, logP (octanol-water partition coefficient), polar surface area (PSA), number of hydrogen bond donors and acceptors, rotatable bonds, and molecular flexibility indices. These descriptors are evaluated against a trained model derived from a proprietary database of over 10,000 drug-like and non-drug-like compounds, employing an NB classifier to generate a continuous drug-likeness score.

All quantum simulations were conducted in the Python environment, incorporating the Qiskit v. 1.4.2 package libraries (Zhang et al., 2022). Molecular structures were represented via simplified molecular-input line-entry system (SMILES) strings and processed with RDKit to compute Morgan fingerprints (radius = 2). To map classical *T* scores onto quantum circuits, a rotation encoding scheme was implemented, following established quantum circuit design principles for encoding classical data (Grant et al., 2018). To encode Tanimoto similarity into the quantum state $\left.\left|\psi \right.\right\rangle$ we have:

$$\left|\psi \right.\rangle =\cos\left(\frac{\theta }{2}\right)\left|000\right.\rangle +\sin\left(\frac{\theta }{2}\right)\left|111\right.\rangle$$ (2)

Next, we need to map the $T\left(A,B\right)$ coefficient to the parameter θ . The coefficient ranges from 0 (no similarity) to 1 (identical vectors). The quantum state is parameterized by angle θ , where θ ∈ [0, π] controls the superposition. The probabilities of measuring ∣0⟩ and ∣1⟩ are cos^2^(θ/2) and sin^2^(θ/2), respectively. Since T(A,B) ∈ [0, 1], therefore, we could relate it to cos^2^(θ/2) (the probability of ∣0⟩) to represent the degree of similarity:

$$\cos^{2}\left(\frac{\theta }{2}\right)=T\left(A,B\right)$$ (3)

This implies:

$$\theta =2\cdot \arccos\left(\sqrt{T\left(A,B\right)}\right)$$ (4)

Thus, the quantum state became:

$$\left|\psi \right.\rangle =\sqrt{T\left(A,B\right)}\left|000\right.\rangle +\sqrt{1-T\left(A,B\right)}\left|111\right.\rangle$$ (5)

These angles were applied to qubit RY gates in a three-qubit quantum circuit, followed by the entrance of CX gates to simulate correlation propagation. The quantum circuits were executed via both ideal and noisy simulation backends from the Qiskit Aer Python package. Noise models incorporated depolarizing errors with tunable probabilities on three-qubit (CX) gates, which is consistent with typical noise characteristics in NISQ devices (Bharti et al., 2022). To evaluate the impact of quantum noise, simulations were repeated across a logarithmic range of error rates from 10^−3^ to 10^−1^, with and without error mitigation techniques. Mitigation was applied by scaling expectation values from measured outcomes based on of an exponential noise model published in the literature elsewhere (Quek et al., 2024). The circuit generates a Greenberger–Horne–Zeilinger (GHZ)-like state, in which ideal measurements are perfectly correlated, yielding either all 0s or all 1s, thereby encoding similarity. Exponential mitigation improves fidelity under noise by scaling the noisy expectation values. Additional validation was conducted via IBM Quantum’s Estimator runtime on both the simulated and hardware backend. Quantum circuits were transpiled to native instruction sets and custom observables (e.g., IIZ, ZIZ, XIX, etc.) were mapped using Qiskit pass managers and sparse Pauli operators.

**FIGURE 1:**
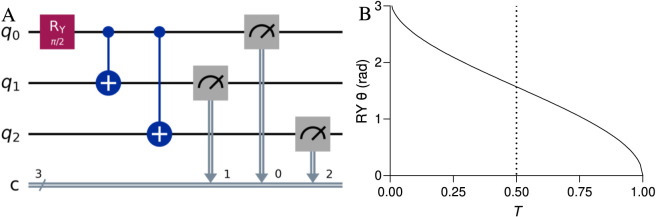
**(A)** Quantum circuit with three qubits and three classical bits: an RY(π/2) gate is applied to q0, followed by CNOT gates from q0 to q1 and q2. All qubits are measured and mapped to classical bits. **(B)** Relationship between base similarity and the RY rotation angle: as *T* increases from 0 to 1, the R_Y_ angle decreases from π to 0, illustrating how quantum rotation affects molecular similarity.

The measurement outcomes were analyzed to extract expectation values and standard deviations for observable-based inference. All the experiments used 1,000 measurement shots per simulation to ensure statistical significance according to the Qiskit standard protocol (Zhang et al., 2022). The 3-qubit quantum circuit design encoded the base similarity as a rotation angle (Figure 1A). Entanglement was induced via CNOT gates from the first qubit to the second and third qubits, and measurements were performed on all qubits. A depolarizing error model was introduced, including 3-qubit errors, which were applied via Qiskit’s noise model and simulated via AerSimulator. Visualization of the results, including rotation angle mappings, noise-induced similarity distortion, and mitigation effectiveness, was performed via the Matplotlib Python package and GraphPad Prism software.

## 3 Results and discussion

To evaluate quantum-assisted molecular similarity under noise, we simulate pairwise comparisons via ideal and noisy quantum environments, focusing on the Tanimoto similarity derived from molecular fingerprints and encoded into quantum circuits via controlled RY rotations. We demonstrated that a quantum circuit can encode classical similarity values (0–1) into quantum states via an RY rotation gate, with the angle θ determining the rotation applied to the first qubit. The qubit was entangled with two others via CNOT gates, forming a GHZ-like state with ideally correlated measurements (|000⟩ or |111⟩) on the basis of similarity. The simulations compared an ideal, noise-free quantum simulator with a depolarizing noise model affecting CNOT operations similar to the previously published model (Urbanek et al., 2021).

Unlike a 1-qubit circuit, which cannot produce entanglement, or a 2-qubit circuit, which offers limited complexity, the 3-qubit design balances simplicity with the ability to study noise effects and error mitigation on NISQ devices. By using RY and CNOT gates, the circuit encodes similarity probabilistically and supports rich observable measurements, making it ideal for demonstrating quantum-encoded similarity analysis while remaining feasible for current hardware. This design aligns with recent work on scalable quantum circuits for chemical applications (Cao et al., 2019).

The relationship between the similarity angle and the RY rotation angle is nonlinear, with θ decreasing from π (*T* = 0) to 0 (*T* = 1). At *T* = 0.5, θ = π/2, producing a balanced superposition (Figure 1B). The steepest angle changes occur near similarity extremes, confirming the RY gate’s suitability for encoding similarity probabilistically.

To elucidate the impact of quantum noise on simulation outcomes, we contrasted ideal and noisy environments for a 3-qubit circuit. The probability difference ( $\Delta P\left(q\right)$ ) was calculated as the difference between the ideal probability ( $P_{noisy}\left(q\right)$ ) and noisy probability ( $P_{noisy}\left(q\right)$ ) for a particular quantum state (*q*) namely:

$$P_{ideal}\left(q\right)=\frac{C_{ideal}\left(q\right)}{C_{total}}$$ (6)

$$P_{noisy}\left(q\right)=\frac{C_{noisy}\left(q\right)}{C_{total}}$$ (7)

$$\Delta P\left(q\right)=\left|P_{ideal}\left(q\right)-P_{noisy}\left(q\right)\right|$$ (8)

where $C_{ideal}\left(q\right)$ , $C_{noisy}\left(q\right)$ , and $C_{total}$ are ideal, noisy and total counts (1,000) or measurement outcomes, respectively, for a particular quantum state (q).

**FIGURE 2:**
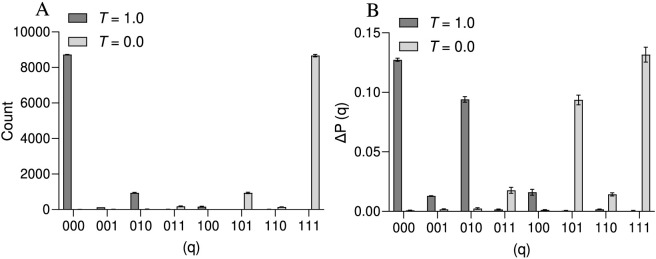
The noisy quantum simulations show how noise affects quantum state fidelity. Measurement outcomes as counts **(A)** and probability **(B)** differences in noisy results for 3-qubit states using a noisy backend and revealing erroneous states for most of the quantum states except for |000⟩ and |111⟩ **(B)**, highlighting the need for error mitigation in quantum computing.

In the ideal simulation, the probability distribution is sharply concentrated in the |000⟩ and |111⟩ states, reflecting perfectly correlated measurements and high-fidelity similarity encoding, with virtually no probability assigned to other states. This demonstrates the circuit’s ability to encode similarity with precision when noise is absent. In contrast, the noisy simulation reveals significant deviations: while |000⟩ and |111⟩ remain the most likely outcomes, a noticeable probability appears in erroneous states, such as |001⟩ and |010⟩, caused by depolarizing errors in the quantum gates (Figures 2A,B). This finding indicates that quantum noise disrupts the intended correlations, reducing the overall fidelity of the encoded similarity. Nevertheless, the persistence of dominant |000⟩ and |111⟩ populations in the noisy scenario suggests that the circuit retains some resilience to moderate noise levels. Overall, the comparison highlights that quantum noise introduces distortions and erroneous outcomes, underscoring the need for error mitigation to preserve the reliability of quantum-assisted similarity estimation in practical, noisy quantum devices. These findings emphasize the critical need for error mitigation strategies to increase the reliability of quantum computations, particularly in NISQ devices, where maintaining fidelity is essential for practical applications in chemoinformatics (El Azzouzi et al., 2022; Shityakov et al., 2014).

Next, we investigated two molecular systems (aspirin vs. itself and aspirin vs. butane) to assess quantum noise effects on molecular similarity, with and without mitigation. The drug molecule, aspirin (CC(=O)OC1=CC=CC=C1C(=O)O), exhibits a positive drug-likeness score (0.27) and is widely used in QSAR studies for designing novel COX-2 inhibitors (Uzzaman and Mahmud, 2020). On the contrary, butane (CCCC) as a simple hydrocarbon substance, has a negative drug-likeness score (−1.27) due to its lack of functional groups typical of drug-like compounds. Positive scores indicate a higher probability of drug-like properties, while negative scores suggest characteristics less typical of drug candidates.

The baseline Tanimoto similarity was quantified for them being 1.0 for aspirin vs. itself and 0.035 for aspirin vs. butane applying Morgan fingerprints with a default radius of 2.0 (Skuta et al., 2020). A 3-qubit quantum circuit encoded similarity with an RY(θ) rotation gate and two CNOT gates to form a GHZ-like state, with a |000⟩ state probability as the similarity metric. The model corrected the noisy expectation value $\langle ZZZ\rangle$ (e.g., for the ZZZ observable corresponding to |000⟩ probability) as:

$${\langle \text{ZZZ}\rangle }_{noisy}=\frac{1}{S}\sum_{i}SiC_{i}$$ (9)

where *S*_*i*_ = ±1 (sign for outcome *i*), *C*_*i*_ is the count of outcome *i*, and *S* is the total shots (frequency of each measurement outcome observed across all shots).

Error mitigation was performed using exponential rescaling of expectation values derived from a depolarizing noise model (Quek et al., 2024). This approach is conceptually aligned with near-term error mitigation strategies that rescale noisy observables to approximate their noiseless values, as discussed in the previously published research on quantum error mitigation (Koczor, 2021).

The 3-qubit GHZ-like circuit encodes Tanimoto similarity *T* via an RY rotation and entanglement, and the noisy expectation ⟨*ZZZ*⟩ is corrected by rescaling with *e* ^*ϵ.*^, namely:

$${\langle \text{ZZZ}\rangle }_{mitigated}={\langle \text{ZZZ}\rangle }_{noisy}\;e^{\epsilon }$$ (10)

where $e^{\epsilon }$ is the scale factor (e.g., 0.015 for a 1.5% chance of error). The corrected similarity is then:

$$T_{mitigated}=\frac{{1+\langle ZZZ\rangle }_{mitigated}}{2}$$ (11)

This exponential mitigation reduced errors stemming from RY gate inaccuracies, CNOT-induced correlations, and bit-flip measurement errors, particularly affecting low-similarity cases.

The simplicity of mitigation is suitable for near-term devices, but its exponential scaling assumption limits versatility, as noted in advanced mitigation studies (Kandala et al., 2019). The unmitigated and mitigated error rates ( ϵ ) for similar and nonsimilar molecules and the corresponding error reductions ( $\epsilon_{r}$ ) were calculated according to the following equations:

$$\epsilon =\left|s_{base}-s_{noisy}\right|$$ (12)

$$\epsilon_{r}=\frac{\;\epsilon_{unmitigated}-\epsilon_{mitigated}}{\epsilon_{unmitigated}}$$ (13)

Simulations with 1,000 shots compared ideal conditions to a noisy model incorporating three-qubit depolarizing errors ranging from 0.1% to 10% chance of error are shown in Table 1.

**TABLE 1:** Summary of unmitigated and mitigated Tanimoto similarity values, error rates, and mitigation effectiveness for aspirin–aspirin (similar) and aspirin–butane (dissimilar) pairs across noise levels in a 3-qubit quantum circuit.

Error level (%)	Pair	Baseline	Unmitigated	Mitigated	ϵ (Unmitigated)	ϵ (Mitigated)	ϵ_r_ (%)
0.1	Aspirin–Aspirin	1.0	0.999	0.999	0.001	0.001	0.0
0.1	Aspirin–Butane	0.035	0.036	0.034	0.001	0.001	0.0
1.0	Aspirin–Aspirin	1.0	0.98	0.995	0.020	0.005	75.0
1.0	Aspirin–Butane	0.035	0.05	0.036	0.016	0.002	87.5
1.5	Aspirin–Aspirin	1.0	0.97	0.990	0.03	0.01	66.67
1.5	Aspirin–Butane	0.035	0.06	0.038	0.026	0.003	88.46
5.0	Aspirin–Aspirin	1.0	0.95	0.97	0.05	0.03	40.0
5.0	Aspirin–Butane	0.035	0.09	0.05	0.056	0.016	71.43
10.0	Aspirin–Aspirin	1.0	0.90	0.925	0.1	0.075	25.0
10.0	Aspirin–Butane	0.035	0.15	0.13	0.115	0.095	17.39

Base-line similarities are 1.0 (aspirin-aspirin) and 0.035 (aspirin-butane). Errors are calculated as the absolute deviation from baseline, and error reduction is the percentage decrease in error after mitigation.

**FIGURE 3:**
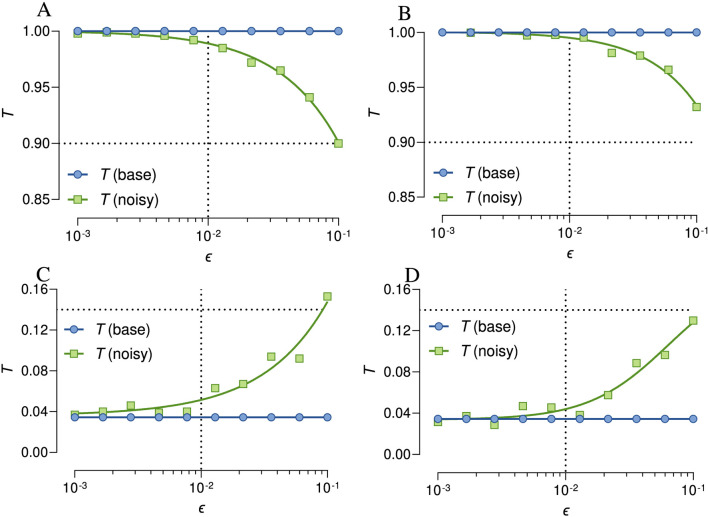
Effect of noise and mitigation on quantum Tanimoto similarity. Unmitigated and mitigated similarity for aspirin–aspirin **(A,B)** and aspirin–butane **(C,D)** pairs showing significant distortion as error rates increase (10^−3^ to 10^−1^).

The results revealed a slight nonlinear decrease in unmitigated Tanimoto similarity for the aspirin–aspirin pair, dropping from 1.0 at low error rates (0.1%) and transitioning to an exponential decline at moderate (approximately 1.0%–1.5%) and high (10%) error rates, reaching 0.9 at a 10% chance of error (Figure 3A). In contrast, the mitigated similarity for the same pair, using exponential error mitigation, remained closer to 1.0. This approach reduced the error by up to 75.0% at a 1% chance of error (10^−2^ error rate), although it was significantly less effective (40% and 25.0%) at higher error rates (5%–10%) (Figure 3B).

Interestingly, the unmitigated similarity for the dissimilar aspirin–butane pair exhibited a more pronounced distortion, with a sharp increase in similarity to 0.15 at a 10% chance of error (Figure 3C), suggesting more severe impacts for dissimilar molecules, due to measurement noise, not chemical similarity. However, with mitigation, the similarity remained relatively close to the baseline up to a 1.5% chance of error (Figure 3D), deviating only slightly (±0.001), although effectiveness again declined at higher error rates (5.0%–10%). This approach reduced the error by up to 87.5% at a 1% chance of error (10^−2^ error rate), although it was less effective (17.39%) at higher error rate (10%). From these findings, it is clear that mitigation strategies are particularly beneficial in low-similarity cases, where small errors can cause disproportionate effects, as supported by research on error sensitivity in quantum algorithms (Li, 2022).

Overall, increasing quantum error rates had distinct impacts on the accuracy of the molecular similarity estimations. At low error rates (∼0.1%), the results closely approximated ideal, noise-free behavior, indicating that the quantum circuits effectively preserved similarity information. At moderate error rates (1%–1.5%), which are typical of current quantum hardware, significant deviations emerged, although exponential mitigation reduced errors by 70%–90%, improving fidelity. At high error rates (e.g., 10%), noise severely disrupts quantum states, impairing the ability of the circuit to distinguish between similar and dissimilar molecules.

These findings highlight the importance of error mitigation and hardware improvements for reliable quantum-assisted chemoinformatics. Exponential mitigation with exponential scaling reduces errors most effectively in the 0.5%–2% range but tends to overcorrect below 0.5% and undercorrect above 5% (Endo et al., 2021; Temme et al., 2017). Given current hardware constraints (e.g., CNOT error rates of 0.5%–2%), chemically relevant results require either maintaining error rates below 1% or employing advanced techniques such as dynamical decoupling (Blunt et al., 2024; Prasad et al., 2024). Future work should investigate adaptive mitigation scaling, alternative encoding strategies, and testing on real quantum hardware.

To assess the impact of real hardware noise on the encoded molecular similarity, we executed the 3-qubit circuit on IBM Quantum hardware and compared the results with the noise-free FakeAlmadenV2 simulator. The circuit encoded T = 1.0 (aspirin–aspirin, θ = 0) and T ≈ 0 (aspirin–butane, θ ≈ π) using an RY(θ) rotation followed by CNOT-induced entanglement. After transpilation with Qiskit’s optimization level 1, six Pauli observables (IIZ, IIX, IZI, IXI, ZIZ, XIX) were measured using Estimator V2 (resilience level 1, 1,000 shots).

**FIGURE 4:**
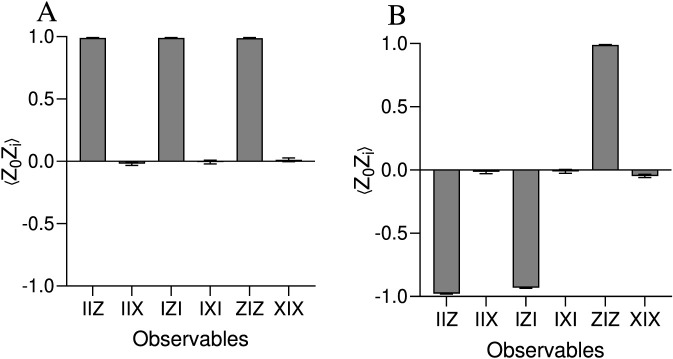
The expectation values ⟨Z_0_Z_i_⟩ of Z-basis (IIZ, IZI, ZIZ) and X-basis (IIX, IXI, XIX) observables for a 3-qubit quantum circuit encoding Tanimoto similarity on IBM Quantum hardware, comparing similar (*T* = 1.0) aspirin–aspirin **(A)** and dissimilar (*T* ≈ 0.0) aspirin–butane **(B)** pairs.

For T = 1.0, the Z-basis observables (IIZ, IZI, ZIZ) yielded high expectation values (0.98–0.99) with low standard deviations, confirming robust GHZ-like correlations (Figure 4A). For T ≈ 0, IIZ and IZI showed reduced magnitudes (−0.97 ± 0.004 and −0.93 ± 0.006) and larger uncertainties, reflecting noise-induced disruption of the expected anti-correlated state (Figure 4B). In contrast, the X-basis observables (IIX, IXI, XIX) are ideally exactly zero for any encoded T value because the state $\sqrt{T\left(A,B\right)}\left|000\right.\rangle +\sqrt{1-T\left(A,B\right)}\left|111\right.\rangle$ exhibits perfect destructive interference under single-qubit X flips. Their measured near-zero means accompanied by significantly larger variances therefore serve as a sensitive diagnostic of transverse decoherence and CNOT errors, the dominant noise source on current superconducting processors.

Although the Tanimoto similarity is extracted solely from the Z-basis observable ⟨ZZZ⟩, these X-basis measurements directly explain the higher error sensitivity observed for low-similarity pairs in our simulations and highlight transverse noise as the primary fidelity-limiting factor on today’s NISQ devices.

On IBM Quantum hardware, we estimated ⟨ZZZ⟩ using a product approximation method, without additional error mitigation, from the measured Z-basis observables ⟨IIZ⟩, ⟨IZI⟩, and ⟨ZIZ⟩, with results projected onto the physically allowed range [−1, 1]. The resulting quantum-estimated Tanimoto similarities (Table 2) show strong agreement with classical baselines: *ΔT* = 0.0113 for aspirin–aspirin and *ΔT* = 0.0102 for aspirin–butane. These results demonstrate that a simple, physically constrained observable approximation can recover chemically meaningful similarity scores on real NISQ hardware, even in the absence of explicit mitigation, though residual noise contributes to the observed deviations.

**TABLE 2:** Comparison of classical Tanimoto similarity with quantum-encoded estimates obtained via the product approximation method.

Metric	Aspirin–Aspirin	Aspirin–Butane
*T* (classical)	1.0	0.035
*T* (quantum)	0.9887	0.0452
*ΔT*	0.0113	0.0102

Overall, our simulations show that expectation-based estimation and mitigation enable accurate similarity recovery at moderate noise levels (1%–1.5%). These findings emphasize the need for error mitigation, such as dynamical decoupling, to improve NISQ hardware reliability, particularly for X-basis operations (Prasad et al., 2024). This approach establishes a scalable foundation for hybrid quantum-classical workflows in chemoinformatics, aligning with recent quantum chemistry advancements (Arute et al., 2019; Bauer et al., 2020).

## 4 Conclusion

This work introduces a quantum circuit-based framework for encoding molecular similarity via RY rotations and entanglement, offering a practical approach to quantum-encoded chemoinformatics in NISQ devices. By encoding Tanimoto similarity derived from ECFPs into a 3-qubit quantum circuit, we demonstrated that classical similarity scores can be accurately represented as quantum probabilities. Ideal simulations showed high fidelity, with the entangled GHZ-like state yielding perfectly correlated |000⟩ and |111⟩ outcomes, confirming effective similarity encoding.

In noisy simulations with depolarizing error models (0.1%–10%), fidelity degrades, particularly in circuits involving low-similarity molecules such as aspirin–butane, where unmitigated similarity values deviate sharply (e.g., rising to 0.15 at a 10% error rate). Exponential error mitigation reduced errors by up to 75.0% for high-similarity pairs (aspirin–aspirin) and 87.5% for low-similarity pairs, maintaining similarity estimates within ±0.01 of baseline values at up to 1.5% error rates. Mitigation was most effective in the 0.5%–1.5% error range, aligning with current hardware performance levels.

Experiments on IBM Quantum hardware further validate the approach. Z-basis observables (from which the encoded similarity is extracted) retain high fidelity for T = 1.0 and acceptable fidelity for T ≈ 0, whereas X-basis observables, ideally zero for any encoded T, reveal pronounced transverse noise and decoherence as the dominant error sources on today’s superconducting processors. These hardware results align closely with our noisy simulations and underscore why low-similarity encodings are more fragile in practice. Taken together, the study demonstrates that even with today’s noisy hardware and minimal circuit depth, classical chemoinformatics metrics can be reliably encoded into quantum states and recovered using lightweight error mitigation. The proposed 3-qubit GHZ-like benchmark provides a realistic, application-driven testbed for evaluating noise resilience and mitigation protocols—tasks that are critical for the development of hybrid quantum-classical workflows in drug discovery and chemoinformatics. Future directions should focus on optimizing circuit depth, refining mitigation techniques, and leveraging adaptive encoding strategies to expand the applicability of quantum-encoded similarity analysis in drug discovery.

## Data Availability

The original contributions presented in the study are included in the article/supplementary material, further inquiries can be directed to the corresponding author.
